# Unpacking the Tumor Protein D52-like Family: Roles in Intracellular Trafficking and Cancer Progression

**DOI:** 10.3390/cells15030252

**Published:** 2026-01-28

**Authors:** Emma L. Dorward, Michael Ortiz, Claire M. Weekley, Kay K. Myo Min, Pascal H. G. Duijf, S. George Barreto, Michael W. Parker, Claudine S. Bonder

**Affiliations:** 1 Centre for Cancer Biology, University of South Australia and SA Pathology, Adelaide, SA 5000, Australia; 2 Department of Biochemistry and Pharmacology, Bio21 Molecular Science and Biotechnology Institute, University of Melbourne, Parkville, VIC 3010, Australia; 3 College of Medicine & Public Health, Flinders University, Bedford Park, SA 5042, Australia; 4 Hepatopancreatobiliary & Liver Transplant Unit, Division of Surgery & Perioperative Medicine, Flinders Medical Centre, Bedford Park, SA 5042, Australia; 5 Australian Cancer Research Foundation (ACRF) Rational Drug Discovery Centre, St. Vincent’s Institute of Medical Research, Fitzroy, VIC 3065, Australia; 6 Adelaide Medical School, University of Adelaide, Adelaide, SA 5000, Australia

**Keywords:** tumor protein D52 (TPD52), tumor protein D53 (TPD53), tumor protein D54 (TPD54), tumor protein D55 (TPD55), cancer, intracellular trafficking, vesicles

## Abstract

There is growing evidence that dysregulation of vesicle-mediated intracellular trafficking pathways leads to the development of various diseases, including cancer. Cancer exploits the intracellular trafficking pathways to modulate the protein flow, alter cell surface protein expression, and drive the hallmarks of cancer progression, such as sustained proliferation signaling and evading immune surveillance. As such, there is increasing interest in understanding the proteins that regulate these processes to better understand cancer biology and to identify novel ways to hinder disease progression. A group of small proteins, known as the Tumor Protein D52 (TPD52)-like family, has been identified and is increasingly recognized for its roles in intracellular trafficking within cancer cells. This family consists of four members: TPD52, TPD53, TPD54, and TPD55. Herein, we review the current literature on the TPD52-like family in cancer and detail the current known cellular functions (e.g., intracellular trafficking roles, lipid biogenesis, cell proliferation, and cell cycle regulation). Overexpression of family members, notably TPD52 and TPD54, has been heavily implicated in tumorigenic roles such as cell migration, invasion, proliferation, and protein–protein interactions. Additionally, there is mounting evidence that this family also has isoform-specific and/or tissue-specific functions, which is of clinical interest. A better understanding of the mechanistic actions of this protein family holds the promise of identifying novel therapeutic targets that exploit the broader multi-target nature of intracellular trafficking regulators to disrupt oncogenic processes.

## 1. Introduction

The intricate interactions that take place to maintain cellular homeostasis are regulated by vesicle trafficking of newly synthesized proteins and cellular secretion mechanisms [1]. These intracellular trafficking pathways are fundamental to many cellular processes that precisely control the direction of proteins within the intracellular space as well as protein expression on, and secretion from, the cell surface [2]. Proteins synthesized within a cell must first undergo sorting to the correct destination to ensure an appropriate function. Therefore, if dysregulation of such pathways occurs, it can be catastrophic to the normal cellular function and can lead to the development of disease, such as Alzheimer’s disease, diabetes, or cancer [3]. When hijacked during cancer progression, changes to proteins expressed at the cell surface or secreted can aid and accelerate the hallmarks of cancer (e.g., sustained proliferative signaling, resistance to cell death, angiogenesis, metastasis, anti-tumor immunity, metabolic reprogramming), contributing significantly to disease progression [4]. Thus, identifying regulators of intracellular trafficking pathways presents a unique opportunity in cancer research, as disruption of these highly regulated mechanisms could potentially lead to a multi-targeted anti-cancer approach.

Over the years, many proteins have been identified to contribute to dysregulated intracellular trafficking during cancer progression, e.g., Rab GTPases and their effector proteins [5]. More recently, the Tumor Protein D52 (TPD52)-like family have been identified as a group of small lipid-binding proteins (140 to 224 amino acids long) with emerging roles in vesicle-mediated trafficking (e.g., exocytosis, Golgi transport, granule secretion, etc.) [6]. The TPD52 family consists of four members (TPD52, TPD53, TPD54, and TPD55), each characterized by highly conserved coiled-coil motifs. In 1995, Byrne and colleagues were the first to document that *TPD52* is overexpressed in approximately 40% of breast carcinomas [7]. Since this discovery, there has been increasing interest in the other family members and their mechanistic roles in cancer progression. As detailed below, elevated protein expression of TPD52 and TPD54 has been associated with more aggressive cancer phenotypes. In addition, these proteins have shown both tissue-specific and isoform-specific roles, which may elucidate different underlying mechanisms in various disease contexts [8,9]. A better understanding of the TPD52 family may identify cancer-specific mechanisms that could guide therapeutic intervention. Importantly, intracellular trafficking directly affects cancer hallmarks, as trafficking determines the expression levels of signaling receptors (e.g., receptor tyrosine kinases and integrins), immune checkpoint proteins, and metabolic regulators at the cell surface. Therefore, abnormal vesicle trafficking or recycling can impact proliferative signaling, invasion, the immune microenvironment, and cell metabolism. Hence, trafficking regulators, including the TPD52-like family, affect multiple oncogenic processes at the same time. In this timely article, we review the reported roles of the TPD52-like family in trafficking mechanisms and cancer progression.

## 2. The TPD52-like Family

The TPD52-like family consists of four proteins with canonical sequences ranging from 140 to 224 residues in length and sharing 41–59% sequence identity: TPD52, TPD53, TPD54, and TPD55 (Table 1). Each protein has several isoforms, featuring insertions, deletions, and/or mutations compared to the canonical sequence. TPD52 was the first member of this family to be identified with elevated expression in breast carcinomas [7].

**Figure 1:**
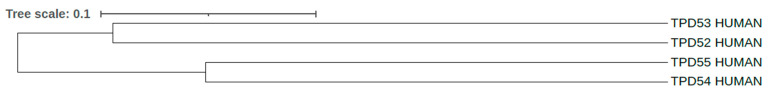
Paralog similarity tree of human TPD52-like family members. FASTA amino acid sequences were obtained from UniProt, and a guide tree was generated using Clustal Omega (https://www.ebi.ac.uk/jdispatcher/msa/clustalo (accessed on 19 January 2026)). Branch lengths represent sequence divergence. The tree scale indicates substitutions per site (scale bar: 0.1 substitutions per site).

TPD52-like proteins are conserved at least across vertebrates, with orthologues found from fish to mammals, as determined by orthology searches using OMA (https://omabrowser.org (accessed on 19 January 2026)). The presence of paralogues across vertebrate species suggests that gene duplication contributed to gene expansion of the family early during vertebrate evolution, followed by divergence of the four human family members. More distant invertebrate homologues may exist, although they are likely to be less readily identifiable. Consistent with this gene divergence, a paralogue similarity tree of the four human TPD52-like family members shows clear sequence separation among the paralogues (Figure 1). This is consistent with functional diversification within the gene family.

**Figure 2:**
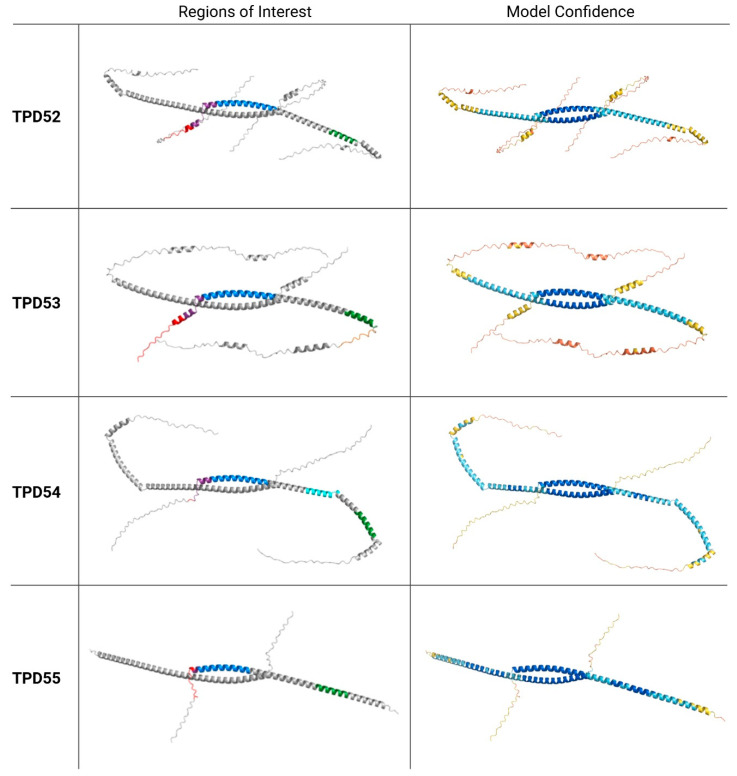
AlphaFold structure predictions of TPD52-like proteins. AlphaFold3 models of homodimeric TPD52-like proteins. Regions of interest are highlighted in one monomer from N- to C-terminal: PEST motifs (red), overlap of PEST and coiled-coil motifs (purple), coiled-coil motifs (blue), insert 2 (TPD54 only, cyan), amphipathic lipid packing sensor (ALPS) motifs (green), and 14-3-3 binding motif (TPD53 only, orange). AlphaFold structure confidence (pLDDT): dark blue = pLDDT > 90 (very high confidence), light blue = pLDDT 70–90 (confident), yellow = pLDDT 50–70 (low confidence), orange = pLDDT < 50 (very low confidence/possibly disordered). Protein structure predictions were generated using AlphaFold3 [10]. Created in BioRender. Bonder, C (2026) https://BioRender.com/clxfx5p (accessed 19 January 2026).

To date, an experimental protein structure has not been determined for any member of the family. These proteins are characterized by coiled-coil motifs ranging from 29 to 52 amino acids (determined with UniProt Align), with TPD55 having the shortest motif. Structural predictions by AlphaFold3 [10] show high confidence in the formation of a coiled-coil domain (pLDDT > 90) in dimeric TPD52-like family models (Figure 2). Outside of this domain, alpha helices and unstructured regions are predicted, with more unstructured regions and reduced confidence in the model (down to pLDDT < 50) nearer to the N- and C-terminals.

The coiled-coil motif was documented to be essential for dimer formation between TPD52-like family proteins, with some contribution from C-terminal regions in facilitating and/or stabilizing the dimers [11,12]. All possible dimeric interactions between these proteins were observed in a yeast two-hybrid system; however, the homodimeric interactions of TPD53 were preferred over heterodimeric reactions, while the opposite was true for TPD52 and TPD54 [11]. Whether the ratio of homodimers and heterodimers formed by TPD52-like family members influences their cellular function remains to be explored.

**Figure 3:**
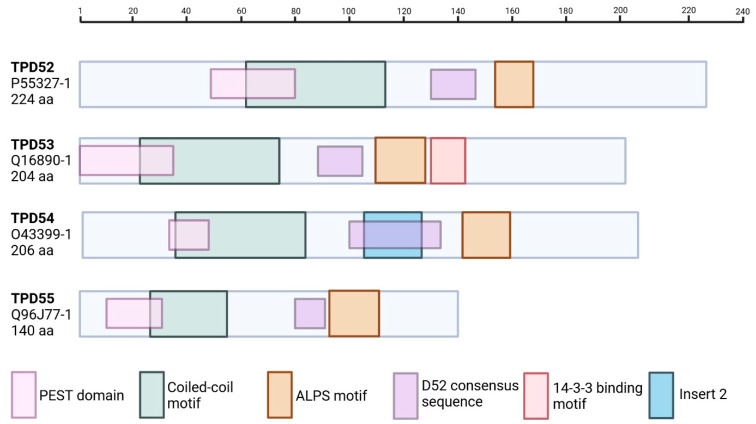
Two-dimensional schematic of TPD52-like protein sequences. TPD52-like proteins are characterized by their coiled-coil motifs (green), biologically relevant PEST domains (pink), and amphipathic lipid packing sensor (ALPS) motifs (orange). Family members also contain a D52 motif (purple); however, the biological function is unknown. TPD53 contains a unique 14-3-3 binding motif (red), whereas TPD54 has a unique insert 2 sequence (blue) [25]. The light blue represents the remaining protein sequence. On the left is the protein name (bold), UniProt ID (canonical sequences used), and protein amino acid (aa) length. PEST sequences were determined by using epestfind (EMBOSS explorer) [15,26]. ALPS motifs determined by sequence similarity and PMIpred [27]. Each dash on the scale bar represents 20aa. Figure adapted from Chen, Frost, and Byrne [25]. Created in BioRender. Bonder, C (2026) https://BioRender.com/clxfx5p (accessed 19 January 2026).

The structural significance of two other motifs in the family is less clear. TPD52, TPD53, and TPD54 share a consensus sequence known as the D52 motif, namely, (V,M)(T,Q)X(T,S) XAY(v,K)KTXETL [13,14], but it does not play a role in interactions between the TPD52-like proteins [14]. PEST motifs overlap with the N-terminal end of the TPD52-like family’s coiled-coil motifs (Table 2 and Figure 3). These motifs are hydrophilic sequences rich in proline, aspartate or glutamate, serine, and threonine residues, with a PEST score (determined by the EMBOSS epestfind algorithm [15]) indicating they are likely biologically relevant proteolytic cleavage sites [16]. C-terminal PEST motifs have also been identified for the TPD52-like family of proteins, but their PEST scores are below the threshold for biological relevance.

Finally, the TPD52-like family proteins contain an amphipathic lipid-packing sensor (ALPS) motif. First identified in TPD54 [17], the highly conserved ALPS motif is also present in TPD52, TPD53, and TPD55, downstream of the coiled-coil motif (Figure 3). Reynaud et al. [18] report that beyond the coiled-coil region, TPD54 is intrinsically unstructured, with the ALPS motif contained in one of several amphipathic helices (Figure 2). These amphipathic helices are expected to fold upon interaction with lipid membranes. Indeed, Reynaud et al. [18] show that there is an increase in alpha helical structure in residues’ C-terminal to the coiled-coil motif upon binding of TPD54 to highly curved, unsaturated lipid membranes. The coiled-coil motif itself is not involved in membrane binding, and monomeric TPD54 can bind to membranes [19]. The protein–membrane interaction is instead facilitated by two amphipathic helices at residues 101–120 and 141–158, the latter of which contains the ALPS motif [18]. Via these interactions of amphipathic helices with lipid membranes, TPD54, TPD53, and TPD52 are able to bind a class of small intracellular transport vesicles. Dubbed intracellular nanovesicles (INVs), these transport vesicles are defined by their association with at least one of the TPD52-like proteins [19].

The TPD52-like proteins have been associated with proteins outside of the family. In the context of INVs, the TPD52-like proteins have been associated with a wide variety of Rab GTPases [19]. Direct interactions have also been reported, and these appear to be mediated through a number of different regions of the TPD52-like proteins [12,20,21,22]. The binding of TPD52 to the integral membrane protein PLP2 and the membrane-associated protein Rab5c was found not to require the coiled-coil motif, but a region that includes the ALPS motif [12]. The formation of a stable TPD52-Activated Protein Kinase (AMPK) 1 complex involves interactions between the AMPK1 α1 and α2 subunits and the N-terminal residues 1–61 of TPD52 [21]. TPD52 interacts with Peroxiredoxin 1 (PRDX1) in prostate cancer cells via C-terminal residues to promote peroxidase activity [22].

Thus, the TPD52-like proteins are versatile in their ability to interact with each other, with heterologous binding partners and with lipid membranes. The TPD52-like protein structures are highly dynamic, with some regions becoming more structured as they interact with other proteins and lipid membranes. The different preferences for forming homo- and heterodimers within the TPD52-like protein family, and the variety of associations with heterologous proteins suggest that some of the functions of TPD52-like proteins are mediated and controlled through an array of protein–protein interactions along their length. Understanding the structure and function of the TPD52-like proteins is further complicated by the existence of multiple isoforms of each TPD52-like protein.

Alternative splicing is believed to be a key feature of the TPD52-like family, with the presence or absence of cDNA inserts possibly determining the protein function and achieving isoform-specific roles (Table 2 and Figure 3) [13]. Of note, the addition or removal of a consensus 14-3-3 binding site via alternative exon splicing has been identified as a novel mode of regulating 14-3-3 binding in TPD52-like isoforms. TPD53, unlike other family members, retains a 14-3-3 binding motif identifying this as a unique protein interaction and exhibiting an integral role in TPD53 protein function [23]. Although, it has been suggested that TPD52 and TPD54 may exhibit neural-specific functions of 14-3-3 binding (due to the presence of a shorter serine-rich exon expressed in neural tissues prior to the 14-3-3 encoding exon), and as such, regulation of 14-3-3 binding with a subset of TPD52-like proteins may be altered in neural tissues [23]. In contrast, sequences designated as insert 2 are restricted to TPD54 isoforms (Figure 3) [13]. Although the function of this insert is not clear, TPD54 isoforms showed stronger interactions with TPD54*+*ins2 isoforms compared to TPD54*-*ins2 isoforms, indicating a role in protein interactions and, consequently, protein function [11]. In addition to insert 2, other inserts (i.e., ins1, ins3 (containing a 14-3-3 binding motif), ins4) are subject to alternative splicing across TPD52, TPD53, and TPD54 isoforms [13]. This diversity of alternative splicing events across the TPD52-like family suggests that various isoforms may modulate TPD52-like protein function. Interestingly, in addition to splicing events, TPD52 was found to be more sensitive to prominent post-transcriptional regulation (particularly by T-cell intercellular antigen 1 (TIA-1) and TIA-related protein binding in the 3′ untranslated region), which affected mRNA stability when compared to other family members [24].

Since their initial characterization, frequent upregulation of the TPD52-like proteins at both gene and protein levels in cancer has been associated with tumor progression and tumorigenesis. For example, Buffart et al. [28] observed a high *TPD52* copy number ratio in liver metastases relative to primary colorectal tumors, supporting a contributory role in cancer progression. In addition to copy number and transcript-level alterations, TPD52-like family proteins have been reported to be increased in absolute protein copy numbers [13,29,30,31]. This high baseline abundance may exacerbate the functional consequences of the gene dosage and overexpression during tumor progression.

Of the four family members, TPD52 and TPD54 are most consistently dysregulated in cancer, and their co-expression has been proposed as a marker for acute myeloid leukemia and acute lymphoblastic leukemia [32].

Building on these observations, the functional relevance of the TPD52-like proteins in cancer is further underscored by their long-recognized involvement in intracellular trafficking pathways. The first heterologous partner identified for TPD52-like proteins was MAL2 (Myelin and lymphocyte protein 2), a proteolipid essential for apically directed protein transport, thereby implicating the family in vesicle trafficking [6]. To better understand the roles of TPD52-like proteins in cellular transport, the following section outlines the key principles of intracellular vesicle trafficking.

## 3. A Brief Overview of Intracellular Vesicle Trafficking

Intracellular vesicle trafficking pathways are integral to a broad range of normal cellular functions, such as protein internalization, secretion, intercellular and interorganelle communication, and signal transduction [33]. These pathways are highly complex and have been the focus of extensive, in-depth reviews in recent years [33,34,35,36,37,38,39]; here, they are discussed briefly.

During the life cycle of a plasma membrane protein, synthesis begins in the endoplasmic reticulum (ER), after which the newly translated protein is transported to the Golgi apparatus for post-translational modifications. The Golgi, a cytoplasmic organelle located near the nucleus, is organized into *cis*, *medial*, and *trans* compartments [40]. Proteins arrive at the *cis*-Golgi network and move sequentially through the *medial* compartment before reaching the *trans*-Golgi network (TGN). At the TGN, fully modified proteins, lipids, and polysaccharides are packaged into intracellular transport vesicles and directed to either the plasma membrane (via exocytosis), early endosomes, or late endosomes [2].

Endosomes are highly dynamic organelles with functionally distinct regions that enable sorting of cargo while simultaneously undergoing protein turnover [41]. They serve as the central trafficking hubs of the cell and are categorized into early endosomes, late endosomes, and recycling endosomes. Increasing evidence suggests that the early endosome and recycling endosome exist in a unified state, often referred to as sorting endosomes, with distinct microdomains enriched in specific proteins or lipids [42]. As a central hub for protein organization, the sorting endosome receives cargo from both the Golgi and the plasma membrane following endocytosis, and subsequently directs it along one of three pathways: a recycling pathway that returns it to the plasma membrane, a retrograde pathway that transports it to the TGN, or degradation via late endosomes [43]. Late endosomes maintain a slightly acidic environment (pH 5–5.5) to prepare cargo for degradation through fusion with lysosomes to form endolysosomes [44]. Lysosomes contain enzymes responsible for digesting unwanted cellular material to facilitate protein degradation.

Autophagy provides an additional degradation pathway targeting cellular debris, damaged organelles, and protein aggregates for recycling through the fusion of autophagosomes and lysosomes, late endosome invagination, or chaperone-mediated pathways [45]. The resulting degradation products (e.g., amino acids) are recycled to support cellular energy production and protein synthesis.

Intracellular vesicles carrying cargo are drawn to the target membrane in part by Rab GTPases. These small proteins serve as master regulators of intracellular trafficking pathways by cycling between an active GTP-bound state and an inactive GDP-bound state [46]. Additional Rab-dependent regulation is exerted through the recruitment of Rab GTPase effector molecules, which include adaptors, phosphatases, motor proteins, kinases, tethers, and regulators of membrane fusion [47]. To date, 66 distinct Rab GTPases have been identified, each associated with specific organelles or trafficking steps to ensure correct cargo delivery [48]. For example, Rab10 is localized to the TGN to regulate TGN-to-plasma membrane trafficking, whereas Rab12 is localized to recycling endosomes and lysosomes to facilitate lysosomal trafficking [46].

Upon arrival at the target membrane, vesicle docking and membrane fusion are mediated by soluble *N*-ethylmaleimide-sensitive fusion protein attachment protein receptor (SNARE) complexes [3]. These complexes are responsible for driving vesicle fusion and exocytosis, and they are necessary for cargo delivery, although they do not provide transport specificity like Rab proteins [49].

**Figure 4:**
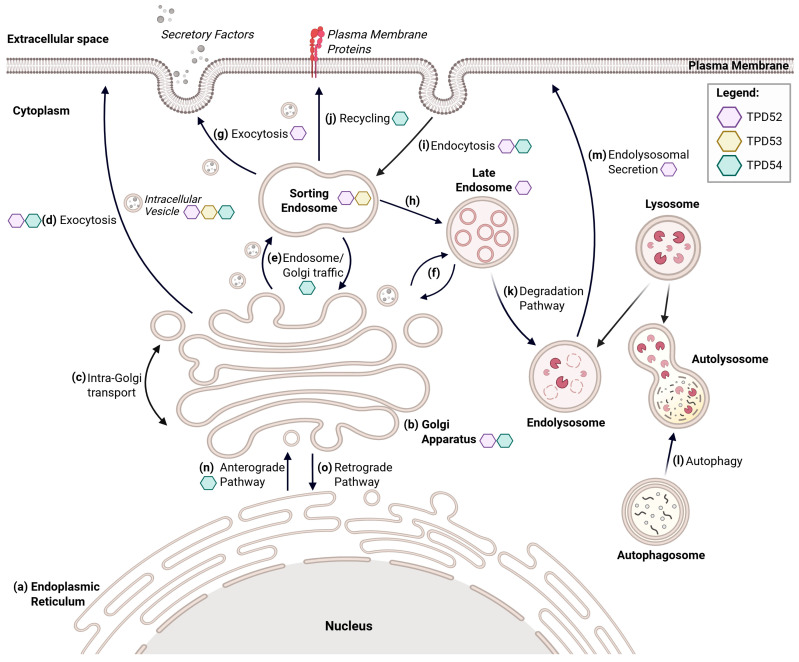
Intracellular vesicle trafficking pathways. Schematic representation of membrane trafficking pathways and major organelles involved (bold), with colored symbols denoting the specific pathways with which each TPD52-like family member is associated. Briefly, proteins are synthesized in the endoplasmic reticulum (**a**), before being transported to the Golgi apparatus (**b**). Once at the Golgi, the proteins undergo intra-Golgi transport (movement of proteins from the *Cis*-Golgi, through the *medial* compartment, then to the *trans*-Golgi network), post-translational modifications, and packaging into intracellular vesicles for trafficking (**c**). Proteins can then be transported to the plasma membrane through exocytosis (**d**), to the sorting endosome (**e**), or to the late endosome (**f**). If directed to the sorting endosome, the protein will be transported either to the plasma membrane for secretion or surface expression (**g**), the late endosome for degradation (**h**), or back to the Golgi apparatus for further modification (**e**). Additionally, proteins can undergo endocytosis at the cell surface and be delivered back to the sorting endosome (**i**) where one of these three fates will be decided. The protein can be recycled and sent back to the plasma membrane (**j**). If sent to the late endosome, fusion with the lysosome will occur, and proteins will be enzymatically degraded (**k**). Lastly, autophagosomes can also collect unwanted proteins or cellular waste and fuse with the lysosome for degradation (**l**). Alternatively, cargo can be released into the extracellular space through endolysosomal secretion (**m**). Transport of molecules toward the cell periphery is termed anterograde transport (**n**), whereas retrograde transport refers to the movement of molecules back toward the cell’s center (**o**). Created in BioRender. Bonder, C (2026) https://BioRender.com/clxfx5p (accessed 19 January 2026).

Proteins that are delivered to the plasma membrane via Rab GTPases and SNARE complexes can then participate in signaling cascades, function as adherence molecules, or undergo secretion. When proteins are no longer required at the cell surface, they are internalized via endocytosis to the sorting endosome, where they are routed to late endosomes or lysosomes for degradation, or towards the plasma membrane or Golgi apparatus for recycling [50]. A schematic overview of these trafficking pathways is shown in Figure 4.

Equally important to the pathways discussed, intracellular transport vesicles are defined by their protein coats. Coat proteins, including coat protein complex (COP) I, COPII, and clathrin, play essential roles in vesicle budding, formation, and functional specificity to ensure correct vesicular-mediated transport. COPII facilitates the formation of vesicles that drive anterograde transport (Figure 4n), carrying newly synthesized proteins destined for secretion or other organelles [51]. In contrast, COPI-coated vesicles mediate retrograde transport (Figure 4o) and support *intra*-Golgi transport (Figure 4c) [51]. Clathrin-coated vesicles participate in endocytosis and transport of lysosomal proteins to the lysosome (Figure 4h,i,k) [43,52]. Collectively, these coat proteins influence vesicle formation, directionality, and, in some cases, cargo selection, thereby shaping the overall architecture of intracellular transport.

Of particular interest, TPD54 has recently been identified by Larocque et al. [29] to bind to the membrane of INVs, a population of highly maneuverable, small transport carriers proposed to function as “express” trafficking routes within the cell [2]. Although the underlying mechanisms of INV biogenesis remain unsolved, TPD54 is required for their function. This raises the possibility that TPD52-like proteins may influence vesicle-mediated transport in a comparable manner to classical coat proteins. Supporting this idea, TPD52 and TPD53 have been observed to partially localize to intracellular vesicle structures, suggesting similar roles shared throughout the protein family [6,53]. Among these, TPD54-vesicle interactions are the best characterized, and the involvement of TPD52-like proteins in INV biology is discussed further in Section 6.

Beyond vesicle association, members of the TPD52-like family have been implicated in multiple intracellular trafficking pathways. Briefly, TPD52 localizes to the Golgi apparatus and to several endosome compartments, including sorting endosomes and late endosomes [54,55]. TPD53 also partially localizes to the early endosomes but primarily has a role in SNARE complex assembly through the coiled-coil domain to participate in membrane fusion events [53]. Similar to TPD52, TPD54 associates with the Golgi apparatus, and it has been reported to maintain Golgi integrity [29]. The protein also interacts with Rab GTPases to support trafficking between the ER, Golgi, and endosomes (anterograde transport), as well as participating in endocytosis and recycling pathways [29]. In contrast, the involvement of TPD55 in intracellular trafficking remains undefined. The trafficking pathways involving TPD52-like proteins are summarized in Figure 4, while the specific functions of each family member are elaborated in their respective sections below.

### Targeting Intracellular Trafficking in Cancer

Dysregulated membrane trafficking is a characteristic of various disease states, such as cancer, where transport pathways are leveraged to execute multiple strategies (e.g., inducing proliferative signaling, halting autophagy, promoting migration and invasion, and secreting enzymes that aid tumorigenesis and contribute to drug resistance) [56]. Although the adaptation of membrane trafficking itself is not classified as a traditional hallmark of cancer [4], it significantly affects many established hallmarks and thus plays a vital role in cancer progression.

At a cellular level, the function of pro-tumorigenic receptors and proteins is intricately linked to their synthesis, processing, and trafficking within the cell. For these receptors to exert their effects, they must first be synthesized in the ER, undergo post-translational modifications in the Golgi apparatus, and be trafficked to the cell surface via vesicular transport as discussed above. Once at the cell surface, they can interact with their ligands or other signaling molecules [57], contributing to processes such as immune escape, tumor angiogenesis, and metastasis. Given that many key pro-tumorigenic proteins rely on this trafficking pathway to function, targeting the machinery that controls their movement to the cell surface presents a potentially transformative approach. By inhibiting key components of this trafficking machinery, it may be possible to prevent the surface expression of multiple pro-tumorigenic proteins simultaneously. This could not only diminish the tumor’s ability to evade immune surveillance but also block other pathways involved in tumor progression, such as angiogenesis, cell survival, and migration. The advantage of such a strategy lies in its potential to target multiple tumor-driving mechanisms at once, reducing the likelihood of resistance compared to single-target therapies.

Recent work increasingly implicates trafficking regulators as active contributors to tumorigenesis rather than passive cellular components, including Apolipoprotein L4 (APOL4) in glioblastoma [58], dysregulated Rab GTPases (e.g., Rab11 in breast carcinomas) [5], and Golgi phosphoprotein 3 (GOLPH3) in breast cancer [59]. This growing recognition, coupled with increasing interest in the therapeutic potential of targeting intracellular transport pathways, places renewed emphasis on vesicle trafficking-associated proteins. Among these, the TPD52-like proteins represent a particularly relevant family, as their trafficking-related functions and dysregulation, both at gene and protein levels, have been increasingly implicated in cancer [29,53,60].

## 4. Mechanistic Functions of TPD52

TPD52 has been strongly implicated in membrane trafficking functions, with protein localization to major trafficking compartments including the Golgi apparatus, early endosomes, late endosomes, and recycling endosomes [54,55]. It has also been reported to reside in close proximity to exocrine secretory granules (i.e., large membrane-bound vesicles), supporting roles in exocytotic pathways [6]. Moreover, TPD52 expression has been found in both the endocytic and exocytic compartments of pancreatic acinar cells, with roles in direct regulation of endolysosomal secretion (i.e., secretion of proteins via the endolysosomal pathway), indirect regulation of secretory granules containing digestive enzymes, and sensitivity to secretagogue stimulation (i.e., molecules that promote secretion by cells) [54,61,62]. Many of these intracellular trafficking roles have been identified via TPD52 binding partners, such as Ca^2+^-dependent binding to annexin VI (a protein implicated in endocytosis), MAL2 (a regulator of intracellular vesicle transport) [54,63], Lysosome-associated membrane protein 1 (LAMP1) (a major component lysosomal membranes that is Ca^2+^-sensitive) [60,64], and Rab5c (localized to early endocytosis and sorting endosomes) [12]. Additionally, through these Ca^2+^-dependent mechanisms, TPD52 expression plays a key role in membrane trafficking during cytokinesis, suggesting a role in cell division [64]. Taken together, these binding partners implicate TPD52 in major protein trafficking pathways both within the cell and for secretion.

Another major role commonly described for TPD52 is its involvement in intracellular lipid storage through lipid droplet biogenesis [55,65,66]. Co-localization with Golgi markers suggests a potential role in transporting lipid droplets directly from the Golgi apparatus, which is consistent with other reported trafficking roles discussed above [25,55]. Through overexpression studies in zebrafish, it has been reported that the AMPK pathway is a primary target of TPD52, leading to lipid accumulation, adipocyte differentiation, and adipose tissue expansion [67]. In addition, TPD52 was found to bind to the alpha subunits of AMPK to form stable complexes (TPD52-AMPK), thereby inhibiting kinase activity and supporting roles in metabolism [21].

### Implications of TPD52 in Cancer

TPD52 is widely reported to be aberrantly expressed in numerous types of cancers, including pancreatic [68,69], breast [70,71,72,73,74,75,76], lung [77], cervical [78,79], ovarian [80], brain [81], bladder [82,83], colorectal [84], prostate [85,86,87,88,89], testicular germ cell tumors [90], and blood cancers [91,92]. This overexpression is primarily due to the increased copy number caused by amplification of the chromosome band 8q21 in which *TPD52* resides [93,94]. When overexpressed in non-malignant mouse fibroblasts (3T3), *Tpd52* induced transformation to a malignant phenotype that promoted tumorigenesis and metastasis [95]. Consistent with this oncogenic activity, Zeng et al. [96] identified TPD52 as a novel deubiquitinating target for Ubiquitin-specific Peptidase 10 (USP10) in gastric cancer cells, enabling TPD52 to evade protein degradation and providing an additional mechanism contributing to dysregulated expression. However, decreased expression of TPD52 has also been reported in renal cell carcinoma [97] and primary hepatocellular carcinoma [98], suggesting that this protein may act as a potential tumor suppressor in these malignancies. These contradictory findings indicate tissue-dependent functions of TPD52 in the progression of cancer, as summarized in Table 3.

Dysregulated TPD52 expression has been implicated in cancer cell proliferation, migration, and invasion in breast cancer [70,107,112,113], cervical cancer [78,79,121,123], melanoma [135], pancreatic cancer [69], oral squamous cell carcinoma [139], and lung squamous cell carcinoma [77]. Many cellular mechanisms have been reported to underlie these pro-oncogenic roles, including PAX-3 (paired box gene 3)-mediated regulation [135], regulation of mitotic checkpoint genes (e.g., Cyclin B1/2, TTK protein kinase, Minichromosome maintenance complex component 4 (MCM4)) [77], involvement in cell signaling axes such as NEAT1/miR-218-5p/TPD52 [112], promotion of epithelial-to-mesenchymal transition (EMT) [107], links to the proliferation-related gene CD58 [91], and suggested sensitivities to hypoxia [139]. In renal cell carcinoma, TPD52 has additionally been reported to act through the phosphoinositide 3-kinase/protein kinase B (PI3K/Akt) pathway to modulate cell proliferation, migration, invasion, and EMT phenotypes [97].

Similar to the tissue-specific function described above, TPD52 has also been reported to exhibit isoform-specific functions. Most notably, *TPD52* amplification is well-documented in prostate cancer, with prostate-specific isoforms (commonly referred to as PrLZ or PC-1) identified as androgen-responsive [86,88]. TPD52 has also been reported to interact directly with the androgen receptor via androgen-response elements in the 5′ untranslated region, contributing to disease progression from androgen dependence to androgen independence (i.e., castration-resistant prostate cancer) [9,149,155]. As such, TPD52 is considered a putative pro-oncogene in prostate cancer progression due to its roles in promoting cell growth, survival, migration, and invasion (regulated through interactions with 14-3-3 proteins and suppression of kinase activity by the tumor suppressor Liver Kinase V1 (LKV1)) [85,87,157,160,166].

TPD52 has been linked to the modulation of various cell signaling pathways involved in cell growth, survival, and metabolism. Multiple studies implicate TPD52 in Nuclear Factor kappa-light-chain-enhancer of activated B-cells (NF-κB), Signal Transducer and Activator of Transcription (STAT) 3, and Akt signaling in cancer cells, all of which are involved in crucial pathways for cellular processes such as cell growth, survival, immune response, and metabolism [102,133,176]. TPD52-mediated activation of the Janus Kinase (JAK)/STAT, PI3K/Akt, and Raf/MEK/ extracellular signal-regulated kinase (ERK) signaling pathways has been reported in neuroblastoma cells to modulate cell differentiation post retinoic acid treatment [138]. This role in cell differentiation has also been reported by other studies, including involvement in the maintenance of neuroendocrine, EMT, and hematopoietic stem cell phenotypes [91,174,180], and B-cell differentiation into plasma cells [101]. This supports early investigations demonstrating that TPD52 expression is involved in the development and maintenance of epithelial cells and embryogenesis [145,184]. Some studies suggest that TPD52 is a downstream target of receptor tyrosine kinase (RTK) signaling, with ERBB2 (also known as HER2) being shown to be co-expressed and have complementary cellular functions with TPD52 to promote breast cancer cell survival [73,75]. Additionally, TPD52 was demonstrated to promote genomic instability through direct interactions with ATM (ataxia telangiectasia mutated) to reduce signaling and, consequently, impair DNA repair capabilities of SK-BR-3 (breast cancer) cells [115]. Genome-wide gene expression analysis of lung squamous cell carcinoma cells supported this finding through the identification of genomic instability as a downstream effect of TPD52 [77]. Taken together, TPD52 appears to promote cell growth and survival through multiple signaling pathways and increases the mutational burden through promoting genomic instability in some cancer cells. Interestingly, there is a reported role for TPD52 in cell stress, with TPD52 driving activating transcription factor 6 (ATF6) activation during ER stress to subsequentially activate the unfolded protein response in liver cancer cells, to promote cell death and ultimately act as a tumor suppressor [82]. Precise mechanisms of TPD52 in these pathways require further elucidation, but the varying reports of a cellular function of the TPD52 protein emphasize its likely context-dependent functions. Additionally, TPD52 has been identified as a downstream target of many different microRNAs to modulate oncogenic mechanisms, as summarized in Table 3 (miRNAs in bold).

As discussed above, TPD52 is primarily reported to be a cell-trafficking protein, a role that extends to its implied roles in cancer progression. It has been reported to bind to annexin VI in a Ca^2+^-dependent manner in both pancreatic acinar cells [63] and myeloma plasma cells [101] to regulate cellular secretory pathways. Through calcium binding, annexin VI plays an important role in membrane interactions and endocytosis, implying that TPD52 may function in intracellular trafficking during oncogenesis [185]. Additionally, TPD52 has been reported to form a complex with Heat Shock Protein Family A Member 8 (HSPA8; a molecular chaperone involved in protein folding, stabilization, and degradation) to activate chaperone-mediated autophagy in prostate cancer cells [163], which serves to aid in protein translocation for cellular recycling and lysosomal degradation. Consistent with this role in trafficking, TPD52 regulates the ER size in response to stress, suggesting potential roles in maintaining ER integrity [82].

In addition to its functional roles, TPD52 has also been reported to be involved in immune cell infiltration in cancer [71]. Through in silico analysis, TPD52 has been associated with the modulation of immune cell infiltration, tumor immune landscape, and immune checkpoints [83,113,183]. There are also a number of reports on TPD52 roles in radio- and chemotherapy sensitivity in triple-negative breast cancer [72] and prostate cancer [158,162,164,175].

In summary, TPD52 is the most well-characterized family member in cancer progression, and although there are varying reports describing either oncogenic or tumor suppressor-like activity, it has well-characterized mechanisms in pro-tumorigenic functions. These functions are likely carried out through intracellular trafficking (including endocytosis, secretion, recycling endosomes, and late endosomes), lipid storage and biogenesis, and cell division.

## 5. Mechanistic Functions of TPD53

Like the other family members, TPD53 has homo- and heterologous binding partners and has been reported to have roles in membrane trafficking, cell cycle regulation, and apoptosis. TPD53 has been shown to partially localize to early endosomes and intracellular vesicle structures [53]. Additionally, TPD53 is involved in the assembly of endosomal SNARE complexes to participate in homotypic or heterotypic membrane fusion as well as the recycling of synaptic vesicles. TPD53 can bind directly to Synaptobrevin 2 (Sb2) via the coiled-coil domain, and Syntaxin (Stx1), to form SNARE-like complexes (Sb2/Stx1/TPD53), which then can go on to participate in membrane fusion [53]. As the coiled-coil domain is a feature shared by all members of the TPD52-like family, it is possible that, if TPD53 functions as a SNARE, the other family members may also possess similar capabilities; however, this is yet to be reported.

TPD53 has been identified as a cell cycle-regulated protein. Protein expression is upregulated at the G2-M transition phase in parallel with cyclin B1 (a known regulator of transition from G2 to M phase) and remains highly expressed into early mitosis (i.e., prophase) before rapidly decreasing at metaphase. As such, dysregulated TPD53 expression in breast cancer cells adversely affects mitosis, leading to the accumulation of multinucleated cells and cell death [186]. Microarray analysis showed that *TPD53* expression fluctuates according to the cell cycle stage in human fibroblasts [187] and HeLa cells [188], as well as being a circadian clock cycling gene in the mouse liver (i.e., involved in the regulation of the daily rhythm of metabolic processes (e.g., lipid metabolism, bile acid synthesis, etc.)) [189]. Moreover, overexpression of TPD53 also occurs during replicative senescence in fibroblasts [190]. Taken together, TPD53 appears to have a role in cell cycle regulation, with dysregulated expression possibly affecting normal cellular division. Supporting this, TPD53 has been reported to undergo alternative splicing events (as discussed above), which allow it to directly bind with 14-3-3, a negative regulator of the cell cycle progression [23]. Other TPD52-like proteins lose this binding motif during alternative splicing (Figure 2 and Figure 3), making this interaction with 14-3-3 unique to TPD53. Following increased TPD53 expression during G2-M phase transition, increased protein binding with 14-3-3 was also observed, suggesting a combined effort to regulate the cell cycle [186]. As 14-3-3 proteins are involved in many cellular processes in both healthy and disease states, this interaction suggests that additional cellular roles for TPD53 are yet to be discovered. Roles for TPD53 in apoptosis have also been demonstrated, with protein expression maintaining apoptosis signal-regulating kinase 1 (ASK1) in a moderately active state to promote the accumulation of pro-apoptotic factors such as caspase [191].

### Implications of TPD53 in Cancer

Unlike TPD52, TPD53 is not overtly implicated in cancer (Table 4). However, a number of reports have described upregulation and involvement in the disease progression of breast cancer [23,186,192], lung adenocarcinoma [193], oral squamous cell carcinoma [194], and colorectal cancer [195]. The limited studies of TPD53 in these cancers include overexpression leading to the modulation of cell viability, migration, and invasion, matrix metalloprotease (MMP) activity, and cell cycle regulation [194,195]. Although the cellular mechanisms are currently unknown, *TPD53* has been identified as having prognostic significance in thyroid cancer [196], colorectal cancers [195,197], hepatocellular carcinoma [198], and nasopharyngeal cancer [199]. Supporting oncogenic functions in these cancers, TPD53 protein expression in patients with ovarian tumors decreased following chemotherapy treatment [200], alluding to some importance in cancer cell survival. Additionally, there is evidence suggesting that TPD53 may have a role in acquired chemotherapy resistance through novel *TPD53-ROS1* fusion rearrangements (fusion of *TPD53* exons 1-3 to *ROS1* exons 33-43) in lung adenosquamous cell carcinoma [201]. Overall, while there is limited evidence that TPD53 functions as a pro-tumorigenic protein in some cancers, the precise mechanisms remain to be elucidated.

## 6. Mechanistic Functions of TPD54

**Figure 5:**
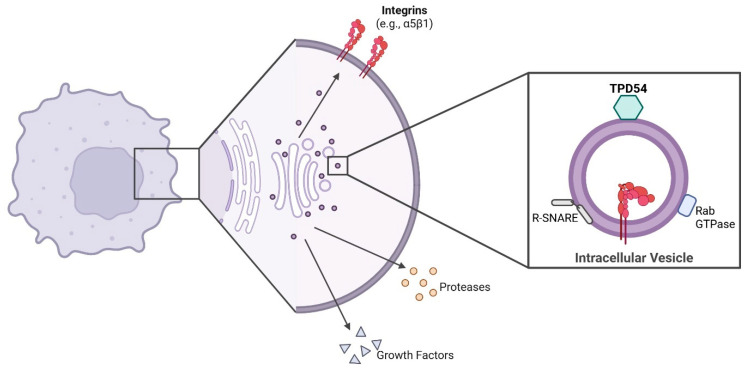
TPD54 in intracellular vesicle trafficking. Schematic of TPD54 binding to intracellular vesicles to transport cargo to the cell plasma [19,29]. Vesicles are drawn to the target membrane by Rab GTPases, and membrane fusion is mediated by SNARE complexes. TPD54 has been reported to have roles in endocytosis and recycling pathways, anterograde trafficking, and Golgi integrity. Created in BioRender. Bonder, C (2026) https://BioRender.com/clxfx5p (accessed 19 January 2026).

The recent literature has characterized TPD54 as a vesicle-trafficking protein that exhibits partial localization to the Golgi apparatus as well as to cytoplasmic compartments [29]. TPD54 exhibits strong associations with Rab14 and Rab2a, and shows a more modest association with Rab5c [29]. Rab14 is important in membrane trafficking between the Golgi apparatus and endosomes, Rab2a is localized to the ER-Golgi pathway, and Rab5c is involved in the endocytic pathway [203,204,205]. These associations implicate TPD54 in multiple cell-trafficking pathways, including anterograde trafficking, endosomal recycling, and the maintenance of Golgi integrity [29]. Among these roles in cell trafficking pathways, TPD54 has recently been identified to be bound to INVs (Figure 5) [29]. As outlined above, INVs are small, highly maneuverable transport vesicles that are hypothesized to provide an efficient transport pathway within the cell [2]. Multiple copies of TPD54 (monomer) can directly bind to the INV membrane via positively charged residues located within the C-terminal region of the TPD54 protein to regulate the trafficking of ligands carried within the INV [2,8]. Residues 83-125, located in the C-terminal domain of TPD54, are responsible for direct membrane binding, while cellular assays showed that positive residues (including K154, R159, K175, and K177) in this region are required for specific binding to INVs in the cell [19]. Interestingly, TPD52—and, to a lesser extent, TPD53—are also associated with INVs and bind independently of TPD54. Larocque et al. [19] reported four INV populations defined by the presence of at least one member of the TPD52-like family (as summarized in Table 5) as well as intermediate populations, but there is evidence that TPD54 is required for correct INV function.

Intracellular vesicles commonly range from 40 nm to 1000 nm, whereas INVs are only 30 nm in size, possibly allowing for fast intracellular transport to rapidly adapt to extracellular cues and precisely control protein flow to and from the plasma membrane [29,206]. INVs have been documented to transport integrins, notably α5β1, with depletion of TPD54 leading to the retention of integrins within intracellular compartments [2,8]. Integrin trafficking has a central role in cell migration and tumorigenesis, suggesting that the TPD54/INV pathway may be altered during cancer progression [2]. Further supporting this, gene enrichment analysis of TPD54 and co-expression genes revealed an enrichment of cell migration pathways and ER-to-Golgi vesicle-mediated transport [207]. Currently, the proteome of INVs is unknown, and it is not fully understood what other cargo they carry. However, TPD54 may act as a major regulator in the intracellular transport of a variety of proteins. Thus, it is tempting to suggest that when TPD54 expression is altered, it may contribute to cancer progression.

### Implications of TPD54 in Cancer

Similar to TPD53, there is limited literature outlining the contribution of TPD54 in cancer (Table 6). However, TPD54 overexpression is linked to pro-oncogenic mechanisms and disease progression in breast cancer [8], clear cell renal cell carcinoma [208], colon cancer [209], glioma [210,211], head and neck squamous carcinoma [207], lung adenocarcinoma [212], pancreatic cancer [213], and prostate cancer [214,215]. In contrast, in oral squamous cell carcinoma [140,216], low TPD54 expression correlates with poor patient outcomes and contributes to oncogenesis. Qiang et al. [217] reported that TPD54 is heterogeneously expressed in glioblastoma with conflicting functions due to its modulation of the Wnt pathway to regulate the EMT status of cancer cells. These findings suggest possible tissue-dependent functions similar to that noted for TPD52.

Dysregulated TPD54 expression in solid cancers has been reported to modulate cell proliferation, migration, invasion, and colony formation. However, the specific mechanisms driving TPD54 involvement in these oncogenic processes are not well understood. In vitro studies demonstrated that TPD54 knockdown inhibited PIK3CA/Akt signaling in pancreatic cancer cells, a pathway that enhances cell survival, growth, and cell cycle regulation [213]. TPD54 inhibition induced cell cycle arrest in the G0/G1 phase, further strengthening its role in cell cycle control [210]. Additionally, TPD54 was shown to reverse tumor-suppressing microRNA activity to promote pro-tumorigenic processes in glioma [211]. Several other studies have also identified novel microRNA molecules that exert tumor-suppressing effects by targeting TPD54 in cancer, further alluding to its importance in solid cancer progression [213,215].

A role for TPD54-driven integrin activation and trafficking during cell attachment and migration in both breast cancer and oral squamous cell carcinoma cells has been reported [8,216], further supporting its involvement in protein trafficking. Further gene enrichment results also found that pathways such as focal adhesion and ER-to-Golgi vesicle-mediated transport were significantly enriched in head and neck squamous carcinoma [207]. This provides preliminary insight into how the TPD54 trafficking pathway may be hijacked during cancer progression to promote cancer cell survival. Further investigation into the role of TPD54 trafficking of other known pro-tumorigenic proteins or growth factors would support this hypothesis.

In silico analysis provided insights into the ability of *TPD54* to modulate immune-cell infiltration (i.e., TAMs and Tregs) in the TME to promote cancer progression [207,212]. These pro-tumor immune cells upregulate the secretion of inflammatory cytokines and chemokines for immune escape. *TPD54* was reported to have associations with key immunosuppressive genes (e.g., *CD274* (*PD-L1*), *EGFR*, *CD44*, *TGF-β1*, and *TGF-βR1*) to further modulate the TME and alter the patient response to treatment in head and neck squamous cell carcinoma and lung adenocarcinoma, highlighting its involvement in tumor immunity [207,212]. Multiple studies have shown a correlation between TPD54 expression and patient sensitivity to traditional treatments (i.e., radiation and chemotherapy) in breast cancers. However, reports of the mechanism of TPD54 in cancer treatment are conflicting. Zhang et al. [8] report that breast cancer patients with high TPD54 tumor expression had significantly shorter survival times after treatment with radiation therapy or targeted molecular therapy, whereas Zhuang et al. [218] reported that TPD54 expression enhanced breast cancer cell sensitivity to metformin treatment via modulation of pyruvate dehydrogenase (PDH) enzyme activity. *TPD54* has also been reported to have prognostic significance in pancreatic cancer patients with early tumor reoccurrence post-surgery [219]. Preliminary studies describe a role for TPD54 in modulating tumor immunity and roles in the treatment response, both of which are of clinical interest and warrant further elucidation.

The current literature provides limited insights into the mechanistic actions of TPD54 in tumorigenic processes; however, it is tempting to speculate that its role in intracellular vesicle trafficking may be as a courier system for cancer cells to hijack and facilitate pro-tumorigenic protein transport to the cell surface. In theory, this would enable the cancer cell to control protein flow and protein recycling, thus aiding cell survival, and ultimately, disease progression. The current evidence for the role of TPD54 in cancer warrants further investigation.

## 7. Mechanistic Functions of TPD55

The least researched member of the TPD52-like protein family, TPD55, was described by Cao et al. [30] to be exclusively expressed in the normal testis, with fertile men having higher expression than infertile men [220]. Interestingly, the adult testis show a 5.6-fold higher expression level of *TPD55* than the fetal testis, supporting a potential involvement in testis development and spermatogenesis [30]. TPD55 was also identified as a novel human sperm tail protein and identified to have roles in sperm capacitation (i.e., physical changes that sperm undergoes in the female reproductive track in order to fertilize an egg) [221]. Although limited, the literature convincingly reports a significant role for TPD55 in the testis and in spermatogenesis.

Interestingly, one of the three known isoforms (isoform 3) was detected in placenta, heart, liver, and adult testis tissue [30]. This aligns with the potential isoform-specific functions identified within this protein family and suggests that isoform- or tissue-dependent functions may be characteristic of the TPD52-like members. TPD55 was also shown to successfully interact with itself and other TPD52-like family members in a yeast two-hybrid system and in vitro [30], likely through the coiled-coil domains characteristic of its family. Thus far, only one heterologous interaction partner has been identified, namely Nuclear Factor I-C (NFIC), which plays roles in cellular differentiation, DNA replication, and gene regulation [222]. Further investigations into this protein are required to fully elucidate its mechanistic actions and significance in cell function.

Utilizing next-generation RNA sequencing, Takematsu et al. [223] reported that *TPD55* was the most downregulated gene in skin samples from type 2 diabetes mellitus patients when compared to samples from non-diabetic patients. To note, additional studies to validate the role of TPD55 in type 2 diabetes are yet to be published, and as such, TPD55 remains widely accepted as a testis-specific protein.

### Implications of the TPD55 in Cancer

Although there is no current literature implicating TPD55 in cancer, it is tempting to speculate a potential role for TPD55 in testicular cancer. Clearly, more research is required.

## 8. Summary and Conclusions

In summary, the TPD52-like family members appear to share several mechanistic aspects in common: coiled-coil domain-mediated homo- and heterodimerization, which regulates vesicle trafficking between the Golgi, endosomes, and plasma membrane. The level and direction of misexpression in cancer, as well as its cancer relevance, typically differ by tissue-of-origin, isoform, and binding context. However, each family member has been associated with the modulation of protein trafficking. This suggests that the cell type and isoform-specific expression patterns determine whether TPD52-like proteins promote or suppress tumorigenesis.

There is increasing evidence that members of the TPD52-like family play critical roles in intracellular trafficking, protein interactions, and cancer progression. Although the precise mechanistic actions are yet to be fully elucidated, the isoform-specific functions driven by alternative splicing may be key to a better understanding of their roles in cancer. The roles that TPD52-like family homo- and heterodimers play in healthy and cancerous cell functions also remain to be answered. However, TPD52 and TPD54 (and, to a lesser extent, TPD53) present themselves as fascinating proteins with roles in intracellular trafficking pathways (e.g., exocytosis, endosome transport, Golgi integrity) and well-documented evidence of overexpression in many cancers. Targeting the TPD52 family as key components of intracellular trafficking pathways offers a single approach to simultaneously prevent the expression of pro-tumorigenic molecules (e.g., integrins) at the cell surface. To this end, studies using cancer vaccines to target tumor self-antigens such as TPD52 have reported improved immune responses against breast and prostate tumors [105,151,224]. In addition, an anti-TPD52 antibody has been identified as a tool for the diagnosis of B-cell malignancies [101]. Moreover, exosomes designed to bind HER2-positive breast cancer cells successfully delivered TPD52-targeting siRNA [117], providing a possible targeted approach to gene therapy. These reports provide confidence in the potential therapeutic benefits of targeting the TPD52-like family to combat cancer.

In conclusion, this review presents evidence that the TPD52-like family has essential roles in cancer cell pathophysiology. Disrupting these intracellular trafficking mechanisms by targeting TPD52/TPD54 in cancer could offer a single approach to interfere with a suite of pro-cancerous cellular mechanisms that underpin cancer progression. Herein, we also identify major gaps in our collective knowledge of the mechanistic actions of these family members that would support TPD52-like based therapies in achieving success in the clinical setting. An important next step will be to elucidate the isoform-specific roles in different tissues and cancer types because alternative splicing may affect family member interactions, trafficking, and downstream signaling. Thus, isoform-specific proteomics and interaction studies will be key for rationally developing therapies that target TPD52-like proteins.

## Figures and Tables

**Table 1:** The TPD52-like family.

Gene Name	*Tumor Protein D52 (TPD52)*	*Tumor Protein D52-like 1 (TPD52L1)*	*Tumor Protein D52-like 2 (TPD52L2)*	*Tumor Protein D52-like 3 (TPD52L3)*
**Alternative names**	Protein Leucine Zipper (PrLZ) Human D52 (hD52) N8 Ca^2+^-regulated heat stable protein (CRHSP-28) Ca^2+^-sensitive phosphoprotein of 28 kDa (CSPP28) Prostate and colon-associated protein (PC-1)	Tumor protein D53 (TPD53) Human D53 (hD53)	Tumor protein D54 (TPD54) Human D54 (hD54) HCCR-binding protein 2	Tumor protein D55 (TPD55) Human D55 (hD55) NYD-SP28
**Chromosome location**	8q21.13	6q22.31	20q13.33	9p24.1
**Amino acid length**	224	204	206	140
**Known isoforms**	8	5	10	3
**NCBI Gene ID**	7163	7164	7165	89882
**UniProt ID**	P55327	Q16890	O43399	Q96J77
**Expression**	Ubiquitous	Ubiquitous	Ubiquitous	Testis

**Table 2:** Residue position of regions of interest in the TPD52-like protein family.

TPD52-like Protein	PEST Motif	Coiled-Coil Motif	ALPS Motif	D52 Consensus Sequence	14-3-3 Binding Motif	Insert 2
**TPD52**	50–80	62–114	151–168	130–143	–	–
**TPD53**	1–36	22–73	111–128	89–103	130–142	–
**TPD54**	34–50	38–82	141–158	100–133	–	106–125
**TPD55**	16–30	28–57	95–112	partial, 83–91	–	–

**Table 3:** TPD52 in cancer. Key: Red—high TPD52 expression correlates with poor patient outcomes, blue—low TPD52 expression correlates with poor patient outcomes, gray—no patient data or animal model investigated, yellow—heterogeneously expressed.

Cancer Type	Role	Reference
**Brain Cancer**	*TPD52* upregulated, with expression increasing as cancer stage advances; identified in gene panels with prognostic or diagnostic value; involved in the protein kinase B (Akt) signaling pathway.	[81]
**Bladder Cancer**	TPD52 promotes activating transcription factor 6 (ATF6) activation during ER stress and regulates ER size; activates the unfolded protein response and increases ER stress-induced apoptosis; APC^Cdc20^ marks TPD52 for degradation through polyubiquitination at K179; acts as a tumor suppressor.	[82]
	Identified in an androgen-responsive gene model for patient prognosis; high *TPD52* expression detected in macrophages and T cells; suggested protective role in immune regulation.	[83]
**Blood cancers**	*TPD52* expression is associated with worse outcomes in infant acute lymphoblastic leukemia.	[92]
	*TPD52* is overexpressed in patients with acute myeloid leukemia and associated with poor prognosis.	[99]
	*TPD52* expression increases in acute myeloid leukemia patients with poor cytogenetic factors; required for hematopoietic stem cell maintenance through regulation of cell proliferation; *TPD52* and *CD59* expression correlate.	[91]
	Lower TPD52 expression is observed in Hodgkin lymphoma patients with bleomycin-induced pulmonary toxicity.	[100]
	TPD52 binds annexin VI in a Ca^2+^-dependent manner in myeloma cell lines; strongest TPD52 expression is detected in plasma cell malignancies; maximal expression occurs during differentiation from B-cells to plasma cells; anti-TPD52 antibody proposed as a diagnostic tool for B-cell malignancies.	[101]
	*TPD52* is frequently co-expressed with *TPD54* in childhood leukemia; decreased *TPD52* expression is associated with hyperdiploid acute lymphoblastic leukemia.	[32]
	Identified as one of the most co-expressed genes with *NFKB1* (encoding the NFκB p50 subunit) in multiple myeloma.	[102]
	Identified as one of eleven proteins activated in ATX-101-sensitive multiple myeloma cell lines.	[103]
**Breast Cancer**	Identified as a potential marker in breast cancer patients carrying a *BRCA1* Ser1841Asn mutation.	[104]
	*TPD52* is significantly overexpressed in patient samples and associated with reduced overall survival; TPD52-expressing fibroblasts exhibit increased proliferation and anchorage-independent growth; reduced TPD52 expression increases apoptosis.	[74]
	TPD52-overlapping synthetic peptide immunization breaks tolerance of TPD52-expressing tumor cells and induces cytotoxic T-lymphocyte activity; TPD52-vaccinated mice show improved survival when challenged with TPD52-expressing breast cancer cells.	[105]
	*TPD52* expression in lymphocytes from breast cancer patients strongly correlates with G2 score.	[106]
	High *TPD52* expression is associated with worse metastasis-free survival; identified as a survival factor in *ERBB2*-amplified breast cancer cells; evidence suggests complementary cellular functions between TPD52 and ERBB2.	[73]
	Higher *TPD52* expression is observed in metastatic tumors; TPD52 is a direct target of **miR-34a**; involved in epithelial-to-mesenchymal transition (EMT), cell invasion, and migration.	[107]
	TPD52 is significantly upregulated; a major target of **miR-107**; miR-107 reduces TPD52 expression and increases sensitivity to paclitaxel.	[108]
	Directly regulated by **miR-449a** and **miR-34a**; further negatively regulated by Star-PAP; involved in cell proliferation and apoptosis.	[109]
	*TPD52* is highly expressed in triple-negative breast cancer tissues; a direct target of **miR-185-5p**; *TPD52* downregulation enhances radiosensitivity of triple-negative breast cancer cells.	[72]
	Identified in a 14-gene, hypoxia-related prognostic signature.	[110]
	*TPD52* is increased in patient blood; expression is elevated in metastatic disease, treatment-naïve patients, and higher tumor stages (III–IV).	[75]
	High *TPD52* expression is associated with poorer survival; it is downregulated by ***miR-101-5p***.	[111]
	TPD52 is upregulated; silencing suppresses cell proliferation and migration; TPD52 knockdown reduces tumor growth in vivo; a target of **miR-218-5p** and involved in an axis with NEAT1.	[112]
	TPD52 is highly expressed and associated with worse survival; a downstream target of **miR-125b-5p** (a cancer repressor); overexpression promotes cell proliferation, invasion, and migration.	[70]
	TPD52 interacts with AMPKα to regulate AMPK activation and cellular metabolism in vitro and in vivo; is positively correlated with lipogenesis and glycolysis gene expression.	[21]
	TPD52 is overexpressed and associated with unfavorable prognosis, particularly in ER^+^/PR^+^/HER2^+^ patients; associated with immune cell infiltration; high-TPD52 patient groups show negative enrichment of signaling pathways including AMPK, fatty acid, cholesterol, glucose, and alcohol metabolism.	[71]
	High *TPD52* expression is associated with lower anti-tumorigenic immune cells; involved in cell proliferation and migration; macrophage migration is significantly decreased following coculture with TPD52 knockdown breast cancer cells.	[113]
	*TPD52* is associated with breast cancer risk; knockdown reduces cell migration and colony formation.	[114]
	*TPD52* expression is increased in patients; highest expression occurs in luminal A/B subtypes; acts as a regulator of Akt and protein kinase C (PKC) pathways.	[76]
	*TPD52* sequence was identified; it is expressed in breast cancer cells and tissues.	[7]
	TPD52 and TPD53 regulate activities through homo- and heteromeric interactions in breast cancer cells.	[11]
	*TPD52* locus represents a target for copy number gain.	[94]
	TPD52 expression in breast cancer cells compromises ATM-mediated cellular responses to DNA double-strand breaks induced by irradiation; direct interactions occur between TPD52 and ATM; TPD52 is suggested as a negative regulator of ATM protein levels.	[115]
	Identified in a panel of genes with alternative splicing and transcriptional events in ER^+^ HER2^−^ and ER^−^ HER2^−^ patients.	[116]
	*TPD52* siRNA delivered by HER2/Neu exosomes to breast cancer cells reduces gene expression by up to 70%.	[117]
	A direct target of **miR-1323**; low miR-1323 expression contributes to TPD52 overexpression and breast cancer progression.	[118]
	TPD52 expression levels increase through activation of estrogen-related receptor alpha (ERRα) pathway.	[119]
	TPD52 is highly expressed in luminal A/B, HER2^+^, and basal-like breast cancer subtypes; a target of **miR-139-5p**.	[120]
**Cervical Cancer**	Direct target of **miR-15a-3p**; TPD52 expression is significantly increased in cancer compared to normal tissue; TPD52 expression decreases after radiotherapy; TPD52 knockdown suppresses proliferation and increases apoptosis in cells exposed to radiation; miR-15a-3p enhances radiosensitivity of cervical cancer in vivo via TPD52 downregulation.	[121]
	TPD52 is upregulated in tissues and cells; hsa_circ_0084927 upregulates TPD52 expression by downregulating **miR-634**; hsa_circ-0084927 inhibition in vivo reduces tumor growth and *TPD52* expression.	[122]
	*TPD52* is significantly upregulated in patient blood; higher *TPD52* expression is observed in early stage (I–II) and non-metastatic patients; patients undergoing chemoradiotherapy show the lowest *TPD52* expression, indicating a better prognosis.	[78]
	Direct target of **miR-218**; elevated expression is associated with increased cell proliferation, migration, and invasion.	[79]
	*TPD52* expression is stabilized by circ_006551 via recruitment of *ELAVL1*; TPD52 overexpression promotes cell migration and invasion capabilities.	[123]
**Cholangiocarcinoma**	TPD52 is elevated in cell lines, tissues, and bile samples; high expression correlates with poorer patient survival.	[124]
**Colorectal Cancer**	*TPD52* is amplified to higher levels in liver metastases compared to corresponding primary tumors.	[28]
	TPD52 is significantly upregulated in all cases of colorectal cancer investigated, irrespective of localization, stage, and grade.	[84]
	*TPD52* is highly expressed in tissue and cells; a downstream target of **miR-139-5p**; TPD52 expression regulated by Krüppel-like factor 7 (KLF7); overexpression of *TPD52* increases cell viability, migration, and invasion; *KLF7* inhibition in vivo reduces TPD52 expression and thereby restricts tumor growth.	[125]
**Gastric Cancer**	Identified as a potential substrate of USP10.	[96]
**Glioma**	TPD52 is negatively regulated by **miR-244-5p**.	[126]
**Glioblastoma**	A target of **miR-103a-3p**; silencing of FGD5-AS1 (FYVE, RhoGEF, and PH domain-containing 5 antisense RNA 1) in vivo leads to decreased TPD52 expression and reduced tumor volume.	[127]
	Exclusively detected in recurrent glioblastoma patient saliva and indicative of relapse.	[128]
**Insulinoma**	Lower TPD52 expression is observed in malignant insulinomas than in benign insulinomas; low expression correlates with poorer patient survival.	[129]
**Lung Adenocarcinoma**	*TPD52* expression is significantly upregulated in tumors; TPD52 expression is higher in brain metastases compared to matched primary lung cancer; possible regulation by **miR-145-5p**.	[130]
	Direct target of **miR-218**; all patient samples stain moderately or strongly positive for TPD52 expression; knockdown of TPD52 decreases cell migration and invasion.	[77]
	TPD52 expression is regulated by the androgen receptor/circ-SLCO1B7/**miR-139-5p** axis; lung cancer patients with lower TPD52 expression have higher survival rates; miR-139-5p directly targets the 3′-untranslated region (UTR) of TPD52 to reduce protein expression.	[131]
	Identified among seven genes coordinately regulated by **miR-145-5p** and **miR-145-3p**.	[132]
	TPD52 is upregulated by circEZH2-mediated sponging of **miR-495-3p**; TPD52 expression activates the NF-κB p65 signaling pathway.	[133]
**Medulloblastoma**	Positive immunohistochemistry staining for TPD52 is a significant predictor of relapse, death, and poor response to chemotherapy.	[134]
**Melanoma**	*TPD52* is identified as a novel *PAX3* (paired box gene 3) target regulating cell proliferation in melanoma cells.	[135]
**Nasopharyngeal Carcinoma**	Higher TPD52 expression is observed in tumor tissue compared to normal tissue; a target gene of **miR-636**.	[136]
	Direct target of **miR-449b-5p** that downregulates expression and thereby suppresses cell proliferation, migration, and invasion.	[137]
**Neuroblastoma**	Expression of TPD52 induces cell differentiation through the Janus Kinase/Signal Transducer and Activator of Transcription (JAK/STAT) signaling pathway; protects cells from apoptosis via Akt and ERK1/2 activation, and arrests cell proliferation by modulating p27^Kip1^ expression.	[138]
**Oral Squamous Cell Carcinoma**	TPD52 mRNA and protein levels increase under hypoxia independent of hypoxia-inducible factor (HIF); TPD52 plays role in cell proliferation and apoptosis under hypoxia; TPD52 knockdown (and inhibition of HIF) in vivo reduces tumor growth.	[139]
	*TPD52* overexpression in TPD54 knockdown cells increases colony formation.	[140]
**Osteosarcoma**	Anti-TPD52 serum reduces cell proliferation and increases apoptosis in vitro; anti-TPD52 serum decreases osteosarcoma growth while increasing cytokine secretion (IFN-γ, TNF-α, IL-12) and apoptosis in vivo.	[141]
**Ovarian Cancer**	TPD52 is significantly overexpressed in ovarian carcinomas while normal and benign tissues are negative for expression.	[80]
	*TPD52* is overexpressed in ovarian cancer compared to normal ovarian tissue and predicts worse survival; a downstream target of **miR-495-3p**; plays a role in cell migration and invasion.	[142]
	*TPD52* expression is increased in patient blood compared to healthy individuals.	[143]
	TPD52 binds to **miR-7515**, and its expression is regulated by long noncoding RNA FTX; this axis facilitates migration, invasion, and EMT.	[144]
**Pan Cancer**	*TPD52* is expressed at higher levels in tumor-derived cell lines from multiple cancers; higher *TPD52* expression is observed in lung tumors compared to normal lung tissue.	[145]
	Identified as one of eight oncogenes upregulated in 258 tumor samples.	[93]
	Low *TPD52* expression correlates with poor survival in breast cancer, papillary renal cell carcinoma, acute myeloid leukemia, liver hepatocellular carcinoma, uterine corpus endometrial carcinoma, and uveal melanoma.	[146]
	*TPD52* is expressed at varying levels in normal tissues; expression is upregulated in 18/29 cancer types, with the strongest *TPD52* upregulation in non-seminoma, ductal, and lobular breast cancer; downregulated in papillary renal cell cancer, leiomyosarcoma, clear cell renal cell cancer, and liposarcoma.	[147]
	TPD52 knockdown significantly alters radiation sensitivity in pancreatic cancer cells (HuP-T3) and cervical cancer (HeLa).	[148]
**Pancreatic Cancer**	High *TPD52* expression is observed in pancreatic cancer cells; *TPD52* silencing reduces cell proliferation, migration, and invasion but induces apoptosis via dephosphorylation of Akt.	[69]
	High TPD52 expression in patients correlates with poorer outcomes.	[68]
**Primary Hepatocellular Carcinoma**	Identified as a potential tumor suppressor; TPD52 expression is decreased in tissues and cells; expression correlates with improved overall survival.	[98]
**Prostate Cancer**	Identification of prostate-specific isoform of the TPD52-like family (*PrLZ* or *PC-1*); amplified expression in prostate cancer is tumor cell-specific.	[88]
	*TPD52* expression peaks at 24 years of age before declining but reactivates during prostate cancer development; expression increases from primary tumor to bone metastasis; expression accelerates cell growth in vitro and in vivo.	[149]
	TPD52 expression improves cancer cell growth, anchorage-independent colony formation, and tumor burden in mice; expression promotes androgen-independent progression and resistance to Casodex; functions in the Akt signaling pathway.	[9]
	TPD52 expression enhances cancer cell proliferation, invasion, and tumorigenicity in vivo; TPD52 may regulate androgen receptor expression and increase prostate-specific antigen (PSA) expression.	[150]
	Mice immunized with a Tpd52 vaccine are protected from prostate tumors that exogenously express Tpd52; vaccination induces a memory cell immune response.	[151]
	*TPD52* overexpression promotes androgen-deprived cancer cell growth, colony formation, and tumor growth in castrated mice; TPD52 prevents release of cytochrome c intracellularly and inhibits androgen-depletion-induced apoptosis; TPD52 elevates phosphorylation of Akt and Stat3 and upregulates Bcl-2 expression.	[152]
	*TPD52* chromosomal region is gained; copy number alterations of *TPD52* correlate with early mortality.	[153]
	Identified to be associated with prostate cancer risk and *HNF1B* expression, a major risk gene for prostate cancer susceptibility.	[154]
	TPD52 can directly interact with the androgen receptor to enhance transactivation in castration-resistant prostate cancer; TPD52 expression increases after androgen deprivation treatment and in castration-resistant patients; silencing TPD52 suppresses tumor growth.	[155]
	TPD52 induces EphA3 expression to promote cell proliferation, survival, and tumor development.	[156]
	*TPD52* is a direct target of **miR-224** regulation; involved in cancer cell migration and invasion.	[157]
	TPD52 expression promotes resistance to rapamycin treatment; TPD52 regulates eukaryotic initiation factor 4E-binding protein 1 (4E-BP1).	[158]
	*TPD52* copy number gain is confirmed in cancer genome; identified in prognostic gene signature that distinguishes five subgroups of prostate cancer.	[159]
	**miR-218** binds to the 3′-UTR of TPD52 to inhibit expression and suppress cancer cell growth.	[160]
	Identified in a 7-gene epigenetic signature (based on methylation status) associated with worse recurrence-free survival; TPD52 methylation inversely correlates with expression.	[161]
	TPD52 expression is upregulated in prostate cancer tissues through the loss of **miR-499a**; TPD52 silencing suppresses in vitro cell proliferation and in vivo tumor growth and metastasis.	[89]
	TPD52 increases docetaxel-mediated drug resistance through interactions with LKB1 to inhibit activation of LKB1/AMPK signals.	[162]
	TPD52 enhances activation of chaperone-mediated autophagy (CMA) through interactions with HSPA8/HSC70; expression is essential for CMA-mediated proliferation and stress resistance; TPD52 undergoes acetylation-dependent regulation to modulate tumor growth in vivo.	[163]
	TPD52 is upregulated in docetaxel-resistant tissues; TPD52 is a direct target of **miR-1182,** and upregulation reduces docetaxel sensitivity.	[164]
	TPD52 downregulation inhibits cancer cell migration and invasion, and reduces tumor growth with a reduction in serum PSA; treatment with an anti-proliferative agent reduces TPD52 expression and slows tumor growth in mice.	[165]
	Direct binding of **miR-103a-3p** to the 3′-UTR of TPD52 reduces expression and inhibits cancer cell migration and invasion.	[166]
	TPD52 can be degraded by E3 ubiquitin-ligase substrate-binding adaptor SPOP; TPD52 degradation is regulated by IL-6 and ERK1/2 phosphorylation.	[167]
	TPD52 expression initiates EMT by activating TGF-β1/p-SMAD signaling as well as downregulating **miR-200** family expression; TPD52-expressing cells lead to the development of metastases in vivo.	[168]
	TPD52 has lower methylation levels in prostate cancer tissues; TPD52 isoform 3 overexpression promotes resistance to mTOR drugs through sustained activation of the Akt pathway and increases phosphorylation of c-Myc and 4E-BP1; TPD52 expression prevents restoration of PTEN by mTOR inhibitors.	[169]
	TPD52 is identified as part of an axis with LINC01122; hypomethylation of the LINC01122/TPD52 axis abnormally upregulates expression and correlates with patient prognosis.	[170]
	*TPD52* is highly expressed in prostate cancer tissues and shows increased copy number; *TPD52* contains androgen-response elements in its upstream promoter, indicating protein level regulation by androgens.	[86]
	Expression of *TPD52* is regulated by upstream negative and positive elements in its promoter region.	[171]
	TPD52 is overexpressed; depletion leads to cancer cell apoptosis and decreases proliferation; expression promotes cell migration via integrin αvβ3 and activation of the Akt signaling pathway.	[87]
	Identification of TPD52 isoforms and their interaction with 14-3-3 proteins.	[172]
	TPD52 is identified as a downstream target of MYST1 and the androgen receptor to regulate cell cycle progression and proliferation of cancer cells.	[173]
	IL-6 administration upregulates TPD52 isoform 1 (PC-1) expression while other isoforms are not affected; TPD52 overexpression enhances neuroendocrine differentiation of prostate cancer cells, which is associated with castration resistance.	[174]
	Depletion of endogenous TPD52 expression increases cancer cell radiosensitivity by reducing DNA double-strand break repair and inducing autophagic-cell death.	[175]
	TPD52 isoform 3 confers transactivation of NF-κB to promote the transcription of TNF-α, IL-6, and IL-8, and it activates STAT3; TPD52 is involved in cell proliferation.	[176]
	*TPD52* isoform (PrLZ) is identified as an androgen-regulated gene in prostate cancer.	[177]
	*TPD52* is significantly upregulated in African American men compared to Caucasian American men.	[178]
	TPD52 interacts with PRDX1 through the C-terminal PEST sequence and regulates peroxidase activity; altered expression of TPD52 affects cancer cell growth, survival, and migration.	[22]
	TPD52 preferentially uses androgen-receptor-induced promoter 2 to generate an alternative first-exon event, resulting in a prostate-specific isoform.	[179]
	TPD52 overexpression inhibits AMPK activation through interactions with LKB1 to exert an oncogenic role.	[85]
	TPD52 isoform 3 induces neuroendocrine differentiation of prostate cancer cells by activation of the STAT3/NF-κB axis; TPD52 positively regulates EMT in cancer cells.	[180]
**Renal Cell Carcinoma**	Decreased gene and protein expression in cells; TPD52 overexpression inhibits cell proliferation, migration, and invasion, and it reduces EMT phenotypes through the PI3K/Akt pathway; overexpression in vivo decreases tumor growth.	[97]
**Salivary Adenoid Cystic Carcinoma**	Higher *TPD52* expression is observed in healthy tissues; TPD52 overexpression significantly suppresses cell migration while knockdown promotes cell migration; TPD52 expression increases epithelial markers; a downstream target of **miR-103a-3p**.	[181]
**Small cell lung cancer**	Axis between TCONS_00020615, **miR-26b-5p**, and TPD52 is associated with small-cell lung cancer.	[182]
**Testicular germ cell tumors**	TPD52 is overexpressed at both gene and protein levels within testicular germ cell tumors compared to normal testis.	[90]
**Uterine Corpus Endometrial Carcinoma**	*TPD52* overexpression is associated with clinical factors and poor survival; implicated in a network with **miR-1-3p**; expression is associated with immune infiltration and immune checkpoints.	[183]

**Table 4:** TPD53 in cancer. Key: Red—high TPD53 expression correlates with poor outcome, blue—low TPD53 expression correlates with poor outcome, gray—no patient data or animal model investigated.

Cancer Type	Role	Reference
**Breast Cancer**	*TPD53* is overexpressed in lymph node-positive breast cancer.	[192]
	14-3-3 proteins are heterologous binding partners of TPD53; alternative splicing regulates 14-3-3 binding to TPD53.	[23]
	TPD53 expression is highly upregulated at the G2-M transition; interactions between TPD53 and 14-3-3 increase in populations enriched for cells in the G2/M phase; TPD53 overexpression produces multinucleated cells, indicating roles in the completion of mitosis.	[186]
**Colon** **Adenocarcinoma**	Identified among a 17-gene, mitosis-related model for predicting survival in colon cancer patients.	[197]
**Colorectal Cancer**	Marker of poor prognosis; *TPD53* expression promotes malignant behaviors in vitro (cell proliferation, colony formation, migration, and invasion); TPD53 knockdown leads to cell cycle arrest in the S phase.	[195]
	Identified in a 9-gene panel found to be significantly upregulated with neoplasia in patients with inflammatory bowel disease (linked to increased colorectal cancer risk).	[202]
**Hepatocellular** **carcinoma**	*TPD53* identified among an 8-gene prognostic model associated with overall patient survival.	[198]
**Lung** **adenocarcinoma**	Case Report: *TPD53-ROS1* fusion variant is identified in a patient that leads to acquired resistance to Osimertinib.	[201]
	Case Report: Novel *TPD53-ROS1* fusion variant is identified in a patient, generated by intra-chromosomal rearrangement.	[193]
**Nasopharyngeal cancer**	Identified as a novel tumor-suppressor gene (among four others) that is downregulated in recurrent nasopharyngeal cancer patients.	[199]
**Oral squamous cell carcinoma**	TPD53 expression promotes cell invasion, migration, and MMP activity via the Akt pathway; *TPD53* overexpression increases cell viability and the percentage of cells in the S phase; *TPD53* overexpression in mice enhances tumor formation and growth.	[194]
**Ovarian Cancer**	Identified to be downregulated in tumors following chemotherapy, with links to acquired chemoresistance.	[200]
**Thyroid cancer**	*TPD53* identified as part of a 6-gene prognostic signature associated with anoikis (apoptosis triggered by loss or improper attachment to ECM) in thyroid cancer patients.	[196]

**Table 5:** Defined intracellular nanovesicle populations.

	First Population	Second Population	Third Population	Fourth Population	Intermediate Populations	
**Bound** **TPD52-like family** **member(s)**	TPD52 TPD53 TPD54	TPD52	TPD54	TPD53	TPD52 and/or TPD54	TPD53 and/or TPD54
**Bound Rab protein(s)**	Rab30 Rab14 Rab1a/b Rab26	Rab10 Rab17	Rab3a Rab4a Rab25	Rab19b Rab33b	Rab11a	Rab12 Rab43

**Table 6:** TPD54 in cancer. Key: Red—high TPD54 expression correlates with poor outcome, blue—low TPD54 expression correlates with poor outcome, gray—no patient data or animal model investigated, yellow—heterogeneously expressed.

Cancer Type	Role of TPD54	Reference
**Breast Cancer**	TPD54 expression decreases sensitivity to radiation therapy; promotes proliferation, migration, and metastasis; mediates α5β1 integrin trafficking during migration; recognizes binding partners TPD52, TPD53, and Rab GTPases.	[8]
	Low TPD54 expression is associated with metformin resistance through modulation of pyruvate dehydrogenase enzyme activity; mitochondrial localization.	[218]
	*TPD54* expression is significantly increased in breast cancer tissue compared to normal breast tissue.	[74]
**Clear Cell Renal Cell Carcinoma**	TPD54 is overexpressed in tumor tissues and is associated with worse survival; expression regulated by DNA methylation; plays a role in negative regulation of immune-cell infiltration; TPD54 knockdown in vitro reduces cell proliferation, migration, and invasion.	[208]
**Colon Cancer**	Identified among four genes that are overexpressed and strongly influence carcinogenesis.	[209]
**Glioblastoma**	Low *TPD54* expression is associated with poor prognosis, enhanced migration, and decreased proliferation rate; regulates EMT status via modulation of the Wnt pathway.	[217]
**Glioma**	TPD54 promotes cell proliferation and colony formation in vitro; knockdown induces G0/G1 cell cycle arrest.	[210]
	Direct target of miR-484-5p; TPD54 expression reverses miR-485-5p-induced repression of cell proliferation, migration, and invasion.	[211]
**Head and Neck Squamous Carcinoma**	*TPD54* is overexpressed and identified as an independent prognostic marker of overall survival; is associated with immune-related signaling, migration, and cancer-related pathways; *TPD54* expression is associated with *TP53* mutations.	[207]
**Lung Adenocarcinoma**	*TPD54* is overexpressed and associated with worse survival; modulates the TME by recruiting TAMs and Tregs; is associated with immunosuppressive genes (*CD274*, *TGF-β1*, and *TGF-βR1*).	[212]
**Oral Squamous Cell Carcinoma**	TPD54 overexpression negatively regulates ECM-dependent cell attachment and migration; decreases integrin activation (inside-out signaling) via modulation of talin1 expression.	[216]
	TPD54 reduces cell migration; overexpression attenuates tumor growth in vivo.	[140]
**Pancreatic Cancer**	TPD54 promotes cell proliferation, invasion, and migration; TPD54 knockdown inhibits PIK3CA/Akt signaling; is a downstream target of miR-217.	[213]
**Prostate Cancer**	TPD54 upregulation is associated with poor prognosis and participates in tumorigenesis and tumor progression.	[214]
	Increased TPD54 expression is associated with increased cell proliferation, migration, and colony formation; miR-503 downregulates TPD54 expression.	[215]

## Data Availability

No new data was created or analyzed in this study.
